# A patient satisfaction survey and educational package to improve the care of people hospitalised with COVID-19: a quality improvement project, Liverpool, UK

**DOI:** 10.12688/wellcomeopenres.17163.2

**Published:** 2022-01-25

**Authors:** Muhammad Shamsher Ahmad, Scott Rory Hicks, Rebecca Watson, Rajia Akter Ahmed, Lewis Jones, Marcella Vaselli, Meng-San Wu, Fatima Hayat, Libuse Ratcliffe, Mark McKenna, Paul Hine, Sylviane Defres, Tom Wingfield

**Affiliations:** 1 Tropical and Infectious Disease Unit, Liverpool University Hospital NHS Foundation Trust, Liverpool, Merseyside, L7 8XP, UK; 2 Clinical Infection Microbiology and Immunology, Institute of Infection and Global Health, Liverpool, UK; 3 Clinical Sciences and International Public Health, Liverpool School of Tropical Medicine, Liverpool, Merseyside, L3 5QA, UK; 4 WHO Collaborating Centre for Social Medicine and Tuberculosis, Department of Global Public Health Sciences, Karolinska Institute, Stockholm, Sweden

**Keywords:** Coronavirus; Covid-19; patient satisfaction; patient perspective; patient feedback; patient experience; quality improvement project

## Abstract

**Background:** The perspectives and experiences of people hospitalised with COVID-19 have been under-reported during the coronavirus pandemic. We developed and conducted a COVID-19 patient satisfaction survey in a large university-affiliated secondary healthcare centre in Liverpool, UK, during Europe’s first coronavirus wave (April-June 2020). The survey found that care was rated highly, including among people of Black Asian and Minority Ethnic (BAME) backgrounds. However, sleep-quality and communication about medications and discharge-planning were identified as areas for improvement.

**Methods:** To improve care for people with COVID-19 admitted to our centre, we designed an educational package for healthcare professionals working on COVID-19 wards. The package, implemented in August 2020, included healthcare worker training sessions on providing holistic care and placement of “Practice Pointers” posters. Patient satisfaction was re-evaluated during the second/third COVID-19 waves in Liverpool (September 2020 - February 2021).

**Results:** Across waves, most (95%) respondents reported that they would recommend our hospital to friends and/or family and rated overall care highly. Comparison of the responses of second/third-wave respondents (n=101) with first-wave respondents (n=94) suggested improved patient satisfaction across most care domains but especially those related to having worries and fears addressed and being consulted about medications and their side-effects.

**Conclusions:** People admitted with COVID-19 to our centre in Liverpool, including those from BAME backgrounds, rated the care they received highly. A simple education package improved the feedback on care received by respondents between the first and second/third waves. These UK-first findings are informing regional strategies to improve person-centred care of hospitalised people with COVID-19.

## Introduction

Severe acute respiratory syndrome coronavirus 2 (SARS-CoV-2) has infected over 171 million people worldwide and caused over 3.5 million deaths ^1^. In the UK, there have been in excess of 4.5 million confirmed cases of whom over 10% have required admission to hospital and more than 152,000 have died ^2^.

Despite extensive research into COVID-19 vaccines, diagnostics, and biomedical treatments, evidence concerning the perspectives of people with COVID-19, especially from vulnerable and Black Asian Minority Ethnic (BAME) groups, is negligible ^3^. To address this, we designed and implemented a satisfaction survey of people with COVID-19 admitted during the first wave of Covid-19 infections (March-June 2020) to our large university hospital in Liverpool, UK. The survey findings (published in Future Healthcare Journal ^4^) showed that nursing and medical care was rated highly and most respondents (96%) reported that they would recommend our hospital to friends or family. However, the survey also highlighted potential areas for improvement including communication about medications and their side effects and informing patients about discharge plans ^4^. To address these shortcomings and improve holistic COVID-19 hospital care, we subsequently implemented a package of complementary interventions on COVID-19 wards in our centre.

Here we report the satisfaction survey findings from the second/third COVID-19 waves in Liverpool (September 2020 - February 2021) and compare them to those of the first wave.

## Methods

This was an unpowered before-and-after observational quality improvement project (QIP) registered with the local Clinical Effectiveness Department. The COVID-19 patient satisfaction survey was developed in collaboration with our centre’s ‘Patient Experience’ team to ensure it was patient-friendly and suitable for people with learning and reading difficulties. According to the policy activities that constitute research at the Liverpool University Hospitals NHS Trust (LUHFT), this Quality Improvement Project met criteria for operational improvement activities exempt from ethics review. All respondents were consented verbally but, in line with local QIP policy, written consent was not deemed to be required ^4^.

The survey was adapted from existing patient satisfaction surveys and integrated with our centre’s “Friends and Family Test” questions. An open-access version of the survey itself and accompanying standard operating procedure can be found in 5.

**Figure 1.**
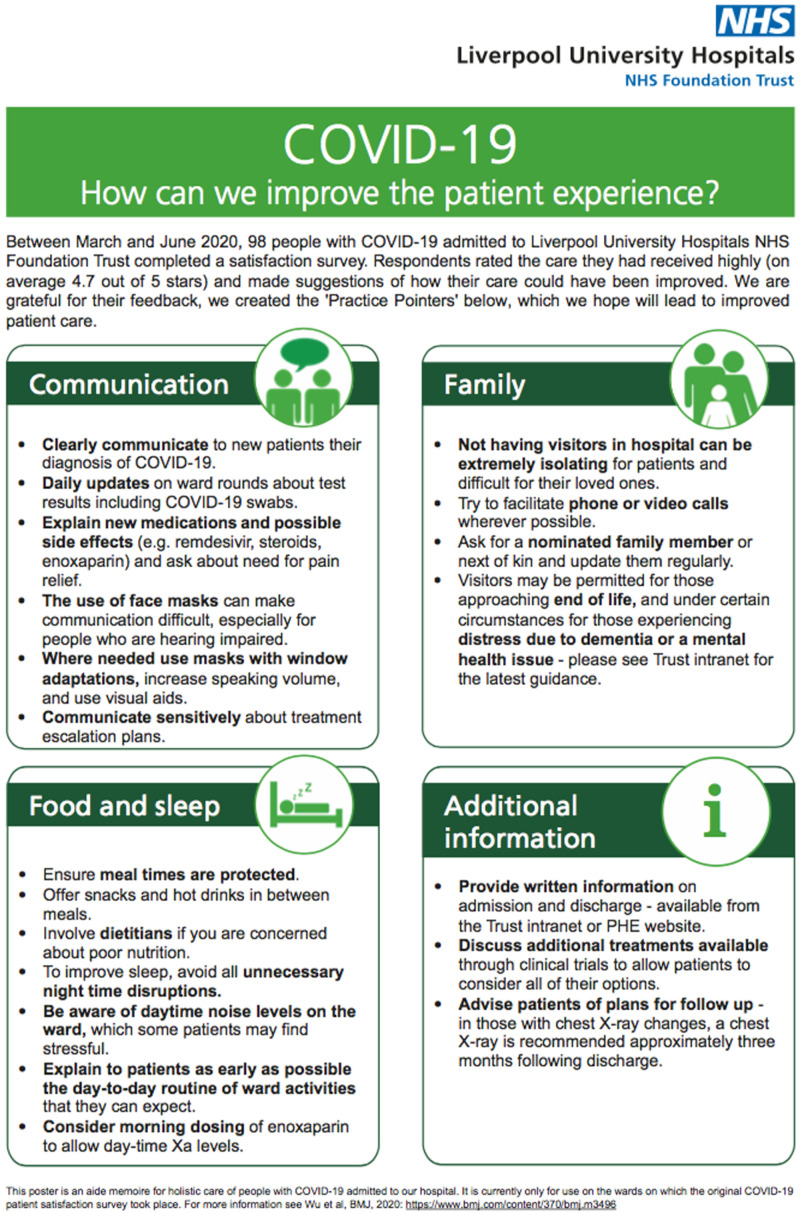
COVID-19 practice pointer poster. Figure 1 showing the COVID-19 practice pointer poster introduced after the patient satisfaction survey during the first wave of pandemic to improve the holistic care services.

The survey was implemented during the first COVID-19 wave (15 ^th^ March 2020 to 15 ^th^ June 2020) and second/third waves (21 ^st^ September 2020 to 6 ^th^ February 2021). Between these time periods, the first-wave survey findings were presented at Tropical Infectious Disease Unit QIP meetings and centre-wide Patient Experience meetings. A package of complementary interventions was designed and implemented in August-September 2021. The package consisted of: concise feedback and training sessions for healthcare workers on holistic care on the COVID-19 wards; updated COVID-19 patient information leaflets for admission and discharge; and a ‘COVID-19 Practice Pointers Poster’ ( Figure 1), which was also placed in visible, shared ward areas.

Descriptive analysis summarised overall responses, compared second/third vs first wave responses, and further compared responses by BAME, age, and gender.

An earlier version of this article can be found on medRxiv (doi: 10.1101/2021.03.23.21253630).

## Results

Surveys from 195 respondents, 94 (48%) from the first wave and 101 (52%) from the second/third wave, were collated ( Table 1). Compared to the first wave, median age of respondents in the second/third wave was higher (64 vs 59 years) and there were more respondents who were female (58% vs 48%), obese (48% vs 38%), active/ex-smokers (49% vs 33%), and had at least one chronic comorbidity (70% vs 67%). The proportion of BAME respondents was the same across both cohorts (10%).

**Table 1.** Sociodemographic and clinical characteristics of survey respondents (n=195).

Variables	Respondents (%)		
	First wave (n=94)	Second/Third wave (n=101)	All (n=195)
**Male**	49 (52)	42 (42)	91 (47)
**Age, median (IQR)**	59 (46-72)	64 (54-73)	62 (50-73)
**BMI, median (IQR)** (n=84/94, 86/101, 170/195) *	28 (25-32)	29 (24-34)	29 (25-33)
**Obesity (BMI ≥ 30 kg/m2, n=84/94,** 86/101, 170/195) *	32 (38)	41 (48)	73 (43)
**Black, Asian and Minority Ethnicity**	10 (11)	10 (10)	20 (10)
**Smoker / ex-smoker**	31 (33)	49 (49)	80 (41)
***Health characteristics***			
Chronic lung diseases **	36 (38)	12 (12)	48 (25)
**Hypertension**	29 (31)	40 (40)	69 (35)
Chronic cardiovascular diseases ***	15 (16)	22 (22)	37 (19)
**Diabetes**	17 (18)	32 (32)	49 (25)
**Chronic Kidney Disease**	13 (14)	16 (16)	29 (15)
**Non-HIV immunosuppression**	9 (10)	7 (7)	16 (8)
**HIV positive**	2 (2)	1 (1)	3 (2)
**History of DVT / PE**	1 (1)	11 (11)	12 (6)
***Number of Comorbidities***			
**0**	31 (33)	31 (31)	62 (32)
**1**	34 (36)	20 (20)	54 (28)
**2**	14 (15)	34 (34)	48 (25)
**3 or more**	15 (16)	16 (16)	31 (16)

Legend: This table shows the number of respondents (%) in the first (n=94), second/third wave (n=101), and overall (n=195). *BMI was only available or calculable for 170/195 respondents because height and/or weight was not documented for 10 respondents in the first wave (n=84/94) and 15 respondents in the second/third wave (n=86/101). **Chronic lung diseases include asthma, COPD, bronchiectasis, interstitial lung diseases, lung cancer. ***Chronic cardiovascular diseases include ischaemic heart disease, heart failure, cerebrovascular disease, and peripheral vascular disease. Abbreviations: IQR interquartile range, BMI body mass index, HIV human immunodeficiency virus, DVT deep vein thrombosis, PE pulmonary embolus

**Figure 2.**
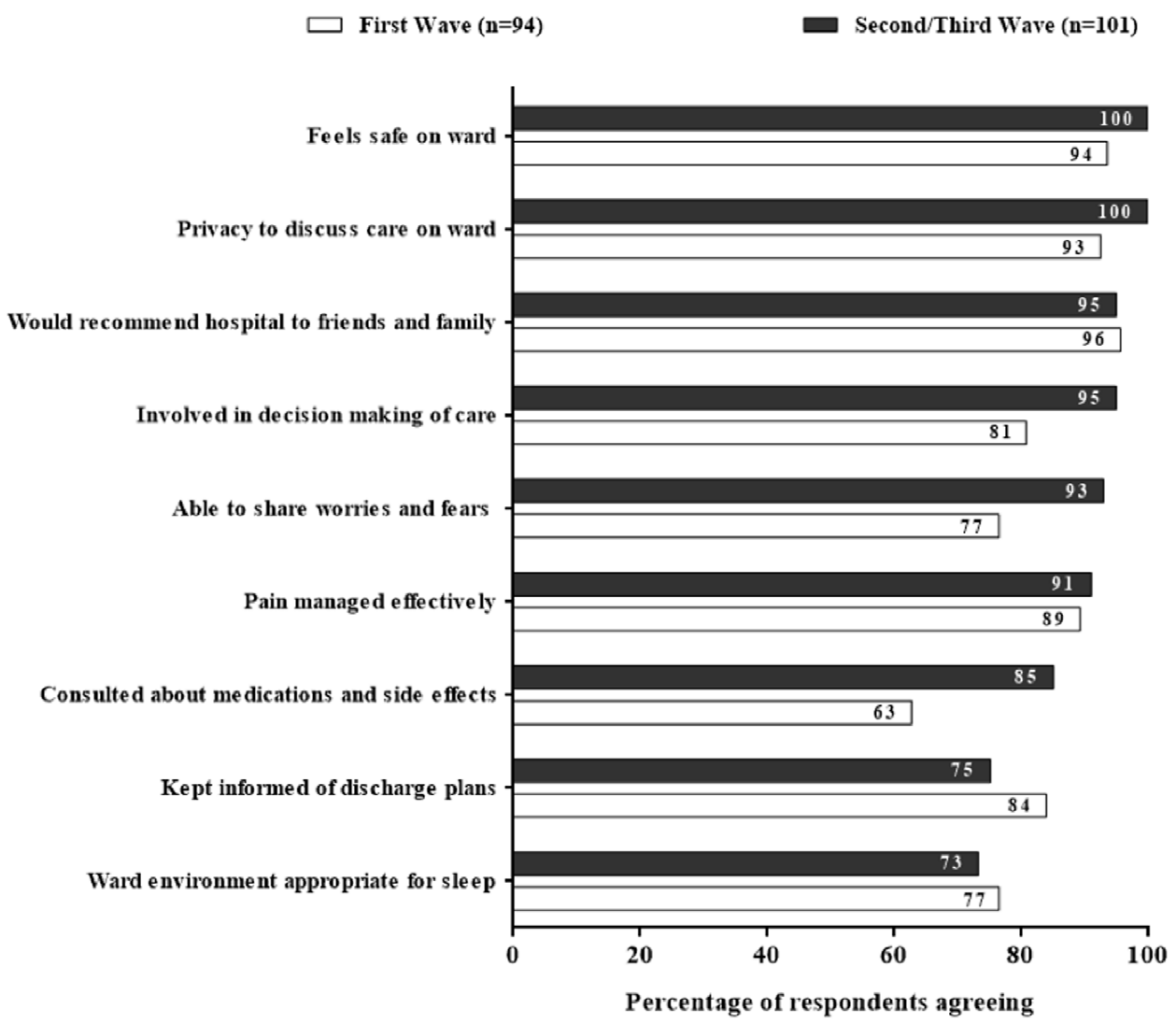
Responses from patients admitted with COVID-19. Figure 2 showing patient satisfaction survey responses (n=195) during the first (n=94) and second-third (n=101) waves of COVID-19 in Liverpool with an overall improvement of satisfaction in most domains after introduction of educational package.

Patient satisfaction was high with overall care rated 4.8/5 on average. Nearly all (95%) respondents reported that they would recommend our hospital to friends and/or family ( Figure 2).

Compared to first wave respondents, second/third wave patient satisfaction increased across multiple domains of care but, most notably, being involved in care decisions (81% to 95%), able to share worries and fears (77% to 93%), and communication about medications and side effects (63% to 85%, Figure 2). Satisfaction decreased with relation to being kept informed of discharge plans (84% to 75%) and sleep environment (77% to 73%, Figure 2).

**Figure 3.**
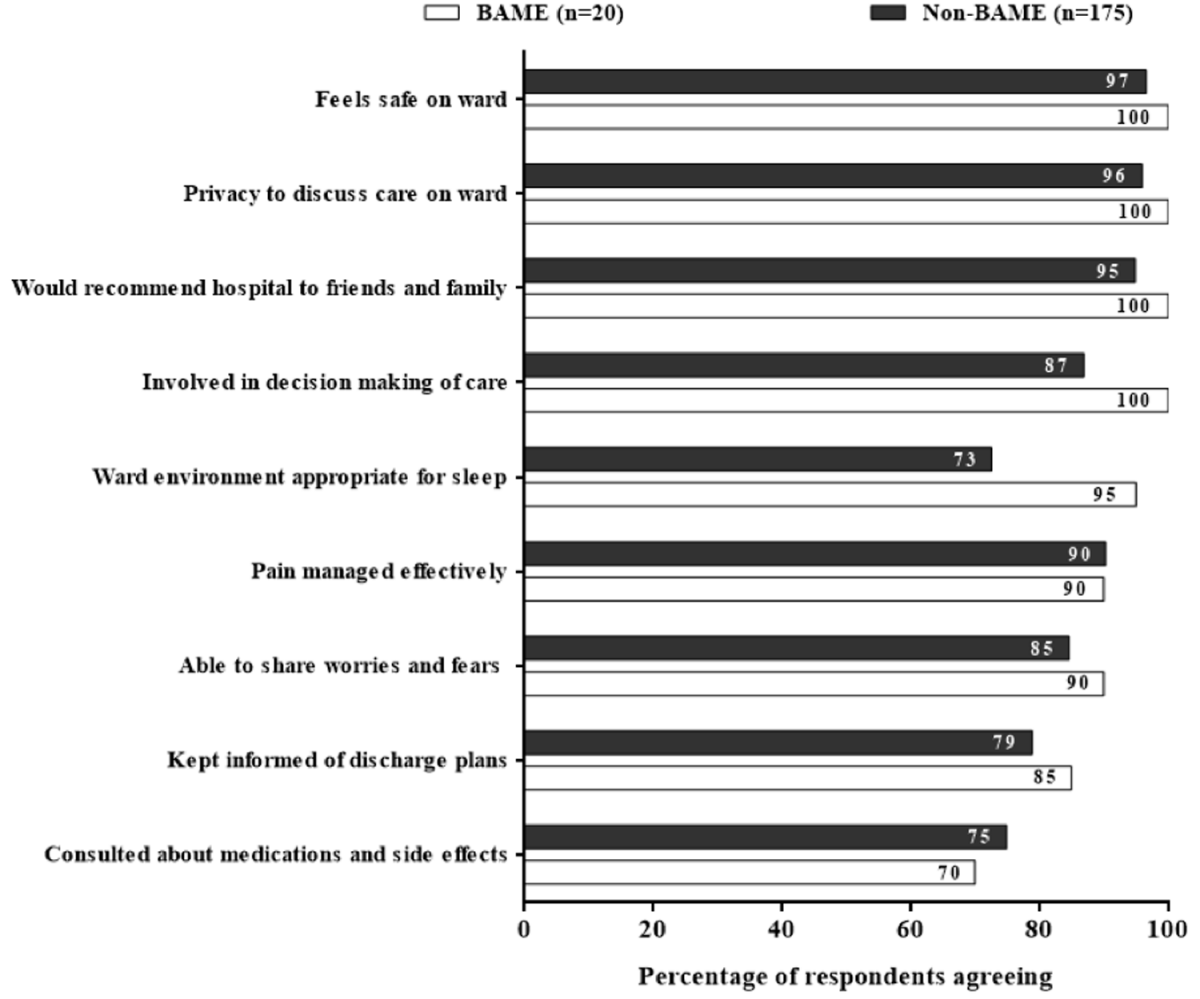
Comparison between BAME and non-BAME Respondents. Figure 3 comparing the patient satisfaction survey responses between the BAME (n=20 and non-BAME (n=175) respondents showing a comparable or even better satisfaction among the BAME patients admitted with COVID-19.

Reported patient satisfaction was higher amongst BAME than non-BAME respondents across all domains except discussion about medications and side-effects (70% vs 75%, Figure 3). Responses were similar by gender and age.

**Figure 4.**
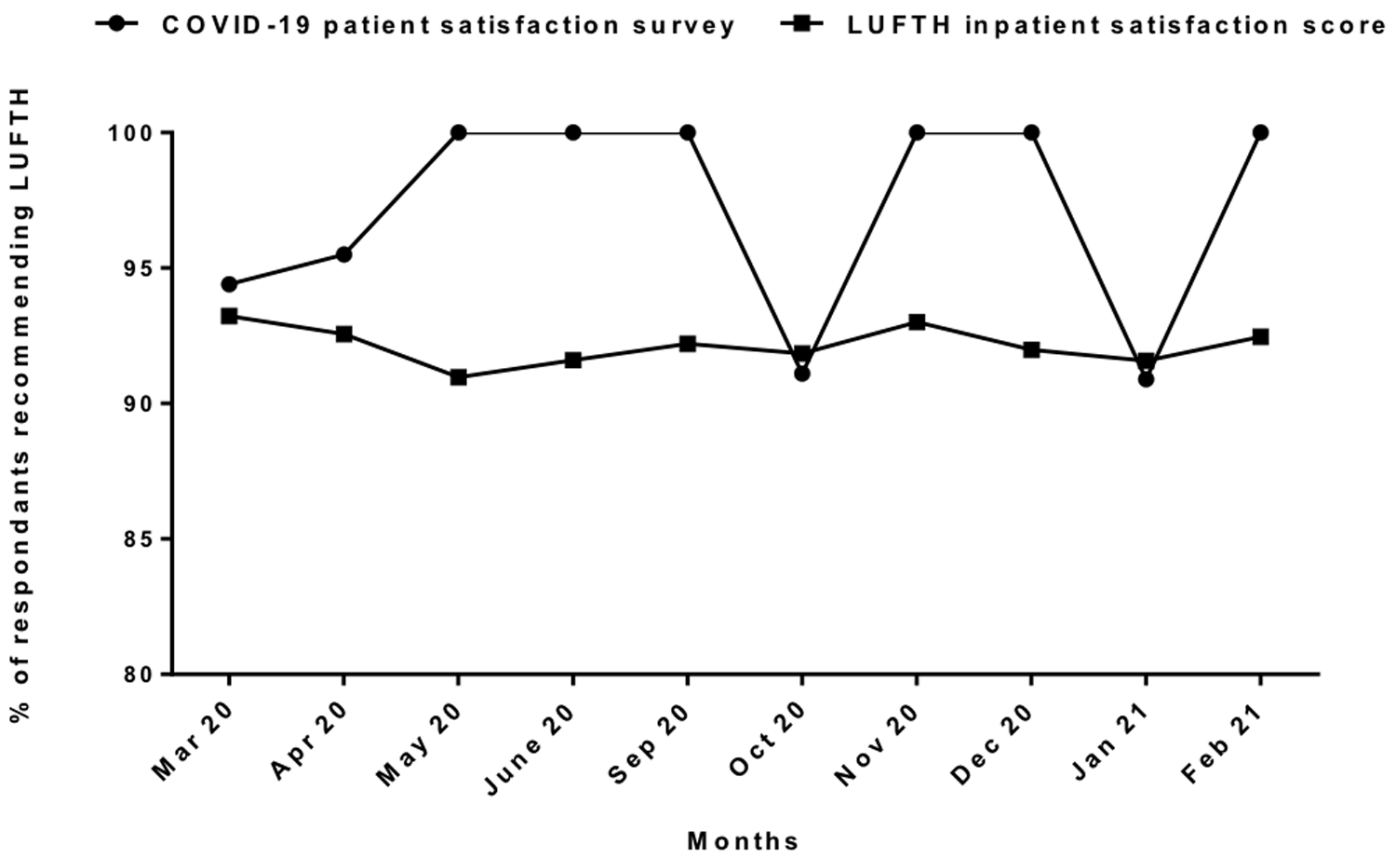
Patient satisfaction between COVID-19 And overall admission. Figure 4 is the month-wise comparison between the overall hospital admissions and the survey participants with COVID-19 admissions during first and second-third wave of COVID-19 pandemic in Liverpool; showing overall better satisfaction in COVID-19 admissions with higher percentage of patients recommending our services in most of the months apart from the two months in the height of second and third waves.

**Figure 5.**
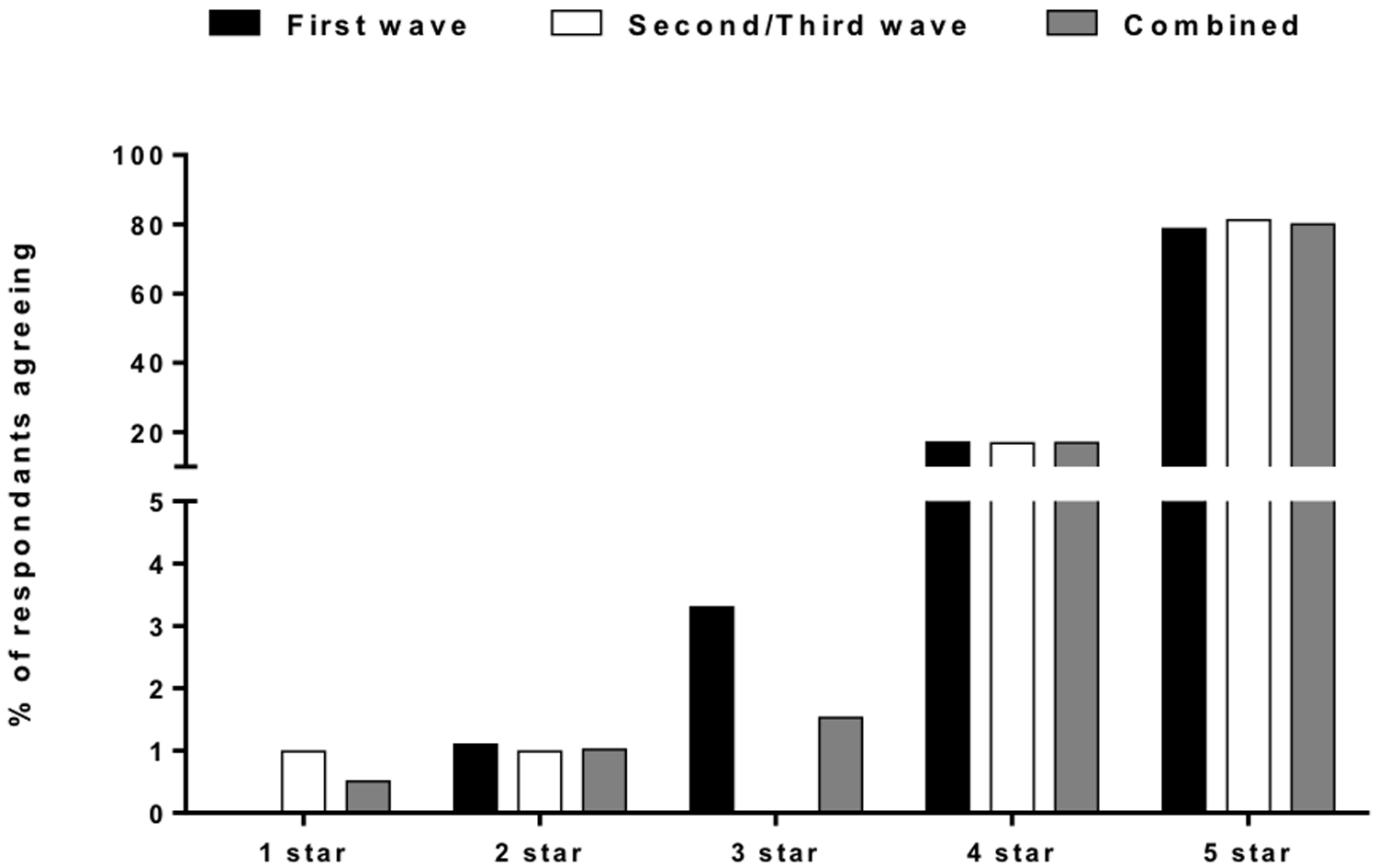
Overall rating of the Hospital Services. Figure 5 showing the rating given to our services by the patients admitted with COVID-19 with nearly all of them rating it above four out of five

Comparison of monthly average “Friends and Family” test responses performed routinely by our centre among all admissions showed that people with COVID-19 were more likely to recommend our centre to friends and family than other patients apart from in October 2020 and January 2021 ( Figure 4). When asked to rate the overall services received out of five, 80% of Covid-19 survey respondents rated it 5/5 and 20% rated 4/5 ( Figure 5).

Free text comments on care received were mostly positive with negative comments focusing on noise level, sleep disruption, and response time when asking for help or pressing patient buzzer ( Table 2).

**Table 2.** Patients’ free text comments during the second-third waves (n=101).

**Things that went well**	Excellent standard of care by all staff. Always someone within seconds when I rang for help. Outstanding staff in all roles. Regular updates to family Allowing family to bring food is very helpful, mentally and physically Couldn’t ask for better Conversation with consultants Most of the staff were great - some exceptional. Food - delicious. Cleaners very thorough at all times. All staff including the cleaners and porters are credit to the NHS Staff are attentive, caring, explained everything nicely Never been in hospital care. Amazing - You are all so good I couldn’t ask for better staff, love them all, made me relaxed away from home
**Things that could be ** **improved**	Don't leave people alone when they are so helpless Ward was very noisy - doors and bins banging, phone ringing unanswered. Had trouble sleeping for outside noise one night Give patients something to pass time (books, TV etc.) Not being able to cope with the pain of the illness

## Discussion

In the UK to date, nearly half a million people have been hospitalised with COVID-19 ^2^. In addition to physical symptoms of COVID-19, those hospitalised can experience negative psychosocial consequences: isolation, including related to infection prevention and control policies; lack of contact with family and friends unable to visit hospital; and uncertainty related to their prognosis ^6^. This may be compounded by constrained communication, trust and rapport with healthcare professionals, particularly for people with hearing impairment due to personal protective equipment including masks ^3, 5, 7^.

Despite these challenges, our COVID-19 patient satisfaction survey showed that the quality of care at our centre was rated highly throughout the COVID-19 pandemic even during the second/third wave when our local health system was under significant operating pressures. While this is the first peer-reviewed COVID-19 patient satisfaction survey of its kind in the UK, a national satisfaction survey of the ‘UK Patient Association’ has been published against which we can compare our findings ^8^. Our survey showed a higher percentage of satisfaction for quality of care and being involved in care decisions. Notably, the 75% of respondents reporting satisfaction regarding discharge planning was consistent with the national survey. Unlike the national survey, the overall satisfaction in patients admitted with COVID-19 remained higher than the general admissions, apart from the dip during the height of the second (October 2020) and third waves (January 2021) in our centre. Keeping in touch with friends and family was identified as a key determinant of inpatient satisfaction in the ‘UK Patient Association’ survey ^9^. Indeed, this was a priority in our Covid-19 Practice Pointers poster ( Figure 1) and the overall improvement of patient satisfaction during the second/third wave in our centre may reflect the positive impact of this practice. Outside of the UK, our findings are broadly consistent with a COVID-19 patient survey carried out in Saudi Arabia ^10^.

Our finding of higher satisfaction amongst BAME respondents across nearly all care domains was encouraging. Compared to non-BAME, people of BAME backgrounds have higher rates of severe COVID-19 disease and death ^11^ and restricted healthcare access ^12^ in the UK. This serves as a reminder of the importance of addressing widening health inequalities related to socioeconomic status and ethnicity in the UK ^11^.

Despite our package of interventions improving some aspects of care ^5^, sleep quality was rated low across both waves. These findings align with pre-COVID surveys and are a persistent issue in hospital care ^13^. There is ongoing work within our centre to address this including noise monitoring and designated “noise free” hours.

Our survey suggested that more respondents with COVID-19 would recommend our centre’s services to friends and family than people admitted for other reasons except in October 2020 and January 2021 – the peak of second and third waves of admissions when our centre was it under the most pressure. However, the overall trend of general patient satisfaction in our centre remained stable during the COVID pandemic as reported in other UK patient experience surveys ^8^.

Almost all of the patients in our survey rated the care they had received during the admission highly. Indeed, COVID-19 respondents in our centre appeared to rate the care they received more highly than those in the UK Patient Association national survey ^9^. 

Although not necessarily reflecting the actual quality or standard of clinical services, patient satisfaction is a popular tool to measure quality of health care services ^14, 15^. A patient survey in the emergency department in New York revealed generally higher level of satisfaction among their attendees during the COVID pandemic compared to the preceding years ^16^. This was attributed in free text responses to gratitude towards the healthcare staff for efforts and sacrifices during the pandemic. The appreciation for the NHS could also be a contributing factor for the positive responses received in our survey.

## Limitations of this study

This was a single-centre, opportunistic, non-randomised survey of a small sample of clinically stable patients, which limits generalisability. Despite this, this peer-reviewed study was a UK first and its findings important when considering person-centred COVID-19 care strategies.

## Conclusions

In a cohort of people hospitalised with COVID-19 in Liverpool, hospital care was rated highly including by those of BAME background. Implementation of an education and training package between COVID-19 waves was associated with improved patient feedback, particularly regarding involvement in and communication about care. The survey and package are being expanded locally to further improve care of people with COVID-19 and other conditions.

## Data availability

Open Science Framework. A patient satisfaction survey and educational package to improve the care of people hospitalised with COVID-19: a quality improvement project, Liverpool, UK. DOI: https://doi.org/10.17605/OSF.IO/7MBJQ ^16^

This project contains the following data:

– Deidentified COVID patient satisfaction survey data 23082021 OSF.xlsx

Data are available under the terms of the Creative Commons Zero "No rights reserved" data waiver (CC BY 4.0 Public domain dedication).
