# Supplementary material for: A patient satisfaction survey and educational package to improve the care of people hospitalised with COVID-19: a quality improvement project, Liverpool, UK
Source: Wellcome Open Res. 2022 Jan 25;6:222. Originally published 2021 Sep 3. [Version 2] doi: 10.12688/wellcomeopenres.17163.2 (PMC8787571; doi:10.12688/wellcomeopenres.17163.2)
Supplement: Peer review history

# Peer review: Reviewer response for version 2

**DOI:** 10.21956/wellcomeopenres.19534.r48262

**Published:** 2022-02-07

**Authors:** Vishesh Paul

**Affiliations:** 1 Pulmonary and Critical Care Medicine, Carle Foundation Hospital, Urbana, IL, USA

Looks good. Strongly consider for indexing.

Is the work clearly and accurately presented and does it cite the current literature?

Yes

If applicable, is the statistical analysis and its interpretation appropriate?

Yes

Are all the source data underlying the results available to ensure full reproducibility?

Yes

Is the study design appropriate and is the work technically sound?

Yes

Are the conclusions drawn adequately supported by the results?

Yes

Are sufficient details of methods and analysis provided to allow replication by others?

Yes

Reviewer Expertise:

Proning in COVID procedural complications in the ICU

I confirm that I have read this submission and believe that I have an appropriate level of expertise to confirm that it is of an acceptable scientific standard.

# Peer review: Reviewer response for version 1

**DOI:** 10.21956/wellcomeopenres.18957.r48161

**Published:** 2022-01-24

**Authors:** Vishesh Paul

**Affiliations:** 1 Pulmonary and Critical Care Medicine, Carle Foundation Hospital, Urbana, IL, USA

I enjoyed reading this manuscript and highly recommend it for indexing. As the article discusses, the majority of the medical community's focus has been on the biomedical treatments of COVID-19. In this, the psychosocial impact of this illness on hospitalised patients may have taken a backseat. Identifying areas of improvement through patient satisfaction surveys such as this one should help us provide more comprehensive and fulfilling care. The points that the team identified, such as discussion about medications and side effects, discharge planning, are being observed globally. Improvement projects such as this one can help guide many centers and providers, especially regarding the care of minorities who often get suboptimal care. 

 I have a couple of suggestions:

1. In the 'conclusion' section of the abstract, it should be BAME backgrounds (not background).
2. Figure 4: Please explain what 'LUFTH' stands for. Readers from outside institutions might not know it. 

Overall, I feel the article is very well written, is succinct, and should be of interest to health care providers and centers worldwide.

Is the work clearly and accurately presented and does it cite the current literature?

Yes

If applicable, is the statistical analysis and its interpretation appropriate?

Yes

Are all the source data underlying the results available to ensure full reproducibility?

Yes

Is the study design appropriate and is the work technically sound?

Yes

Are the conclusions drawn adequately supported by the results?

Yes

Are sufficient details of methods and analysis provided to allow replication by others?

Yes

Reviewer Expertise:

Proning in COVID procedural complications in the ICU

I confirm that I have read this submission and believe that I have an appropriate level of expertise to confirm that it is of an acceptable scientific standard.

# Response

**Published:** 2022-01-24

**Authors:** Tom Wingfield

**Affiliations:** Liverpool School of Tropical Medicine, UK

Dear Dr Paul,

 Thank you for your constructive and supportive comments.

 We have made the updates suggested.

 Yours sincerely,

 Tom Wingfield (on behalf of the co-authors)

# Peer review: Reviewer response for version 1

**DOI:** 10.21956/wellcomeopenres.18957.r46861

**Published:** 2021-11-11

**Authors:** Katherine Ajdukiewicz

**Affiliations:** 1 Department of Infectious Diseases & Tropical Medicine, North Manchester General Hospital, Manchester, UK

Well written and well executed study. Most limitations identified - excludes those who were extremely ill and either died or went to critical care; pre-dates vaccination which may alter future similar studies (with use of ronapreve for example) in spike protein antibody negative patients.  Relevant study with relevant findings and easily reproducible in other settings with realistic interventions. It would be interesting to see if these outcomes are maintained over time and in non-COVID patients.

Is the work clearly and accurately presented and does it cite the current literature?

Yes

If applicable, is the statistical analysis and its interpretation appropriate?

Yes

Are all the source data underlying the results available to ensure full reproducibility?

Yes

Is the study design appropriate and is the work technically sound?

Yes

Are the conclusions drawn adequately supported by the results?

Yes

Are sufficient details of methods and analysis provided to allow replication by others?

Yes

Reviewer Expertise:

Infectious diseases clinician treating patients with COVID-19 since 2020 (in addition to patients with other infections).  Most research is on other areas - bacterial meningitis RCT with adjuvant therapy, CSF and plasma cytokine studies, perinatal HIV cohort studies

I confirm that I have read this submission and believe that I have an appropriate level of expertise to confirm that it is of an acceptable scientific standard.
